# Acoustic Constraints and Musical Consequences: Exploring Composers' Use of Cues for Musical Emotion

**DOI:** 10.3389/fpsyg.2017.01402

**Published:** 2017-11-10

**Authors:** Michael Schutz

**Affiliations:** Music, Acoustics, Perception, and Learning Lab, McMaster Institute for Music and the Mind, School of the Arts, McMaster University, Hamilton, ON, Canada

**Keywords:** music, language, corpus analysis, emotion, communication, composers, acoustics

## Abstract

Emotional communication in music is based in part on the use of pitch and timing, two cues effective in emotional speech. Corpus analyses of natural speech illustrate that happy utterances tend to be higher and faster than sad. Although manipulations altering melodies show that passages changed to be higher and faster sound happier, corpus analyses of unaltered music paralleling those of natural speech have proven challenging. This partly reflects the importance of modality (i.e., major/minor), a powerful musical cue whose use is decidedly imbalanced in Western music. This imbalance poses challenges for creating musical corpora analogous to existing speech corpora for purposes of analyzing emotion. However, a novel examination of music by Bach and Chopin balanced in modality illustrates that, consistent with predictions from speech, their major key (nominally “happy”) pieces are approximately a major second higher and 29% faster than their minor key pieces (Poon and Schutz, 2015). Although this provides useful evidence for parallels in use of emotional cues between these domains, it raises questions about how composers “trade off” cue differentiation in music, suggesting interesting new potential research directions. This *Focused Review* places those results in a broader context, highlighting their connections with previous work on the natural use of cues for musical emotion. Together, these observational findings based on unaltered music—widely recognized for its artistic significance—complement previous experimental work systematically manipulating specific parameters. In doing so, they also provide a useful musical counterpart to fruitful studies of the acoustic cues for emotion found in natural speech.

## Introduction

Music's powerful ability to convey emotion has long served as a point of intrigue, captivating audiences and eliciting introspection from a range of scholars. Charles Darwin once posited that this ability is derived in part from the simplistic vocalizations of our evolutionary ancestors (Darwin, 1872), and musicians such as the renowned conductor/composer Leonard Bernstein frequently looked to language as way of understanding music's communicative power (Bernstein, 1976). Such speculation has led to considerable experimental research on **music–langauge parallels** in the communication of emotion (Scherer, 1995; Mithen, 2005; Coutinho and Dibben, 2013), with broad implications for cognitive science, music cognition, linguistics, and anthropology.

KEY CONCEPT 1 **Music–language parallels.**

These forms of communication share commonalities in their structure and processing, including acoustic cues for emotion. Pitch height and timing are important cues distinguishing happy from sad music as well as speech utterances. In contrast, modality is also an important cue for music, but has no counterpart in speech.

To contribute to this topic, my colleagues and I explore the natural use of emotional cues in music widely recognized for its artistic significance. This *Focused Review* highlights findings from our previous *Frontiers* article documenting that Bach and Chopin use pitch and timing cues in a manner paralleling natural emotional speech (Poon and Schutz, 2015). This manuscript summarizes those findings, clarifying connections with cognate fields ranging from neuroscience and communication to music performance and musicology. Additionally, it places them in context by discussing previous work motivating that study—a survey of musical repertoire exploring the emotional constraints/affordances of particular instruments (Schutz et al., 2008). Together, these findings provide insight into the communication of musical emotion based upon the artistic intuitions of highly trained musicians—complementing approaches using melodies created or manipulated primarily for scientific purposes.

Cues such as pitch height and timing play a powerful role in conveying emotion. For example, when gauging the response of friends to a bold new idea, hearing “that sounds great” holds a different meaning when emitted as high pitched squeal of delight vs. a low groan of dismissal. We are implicitly sensitive to speakers' tendencies to use higher voices (Scherer, 2003) and faster rates of attack (Scherer, 1995; Banse and Scherer, 1996; Sobin and Alpert, 1999) when pleased. Slowly articulated passages are unlikely to sound happy—whether experienced as a spoken utterance or a musical melody (Juslin and Laukka, 2003), and speakers are more affected by sad topics when speaking more slowly (Siegman and Boyle, 1993). For example, imagine the difference in a friend's voice when discussing a promising first date or long awaited sporting victory vs. bemoaning a disappointing encounter or match lost. These same acoustic cues play a crucial role in musical emotion, where higher (Hevner, 1937; Ilie and Thompson, 2006) and faster (Dalla Bella et al., 2001; Gagnon and Peretz, 2003) melodies sound happier than lower and slower ones.

Previous research demonstrates that manipulations of controlled monophonic lines such as raising a melody's pitch (Hevner, 1937; Ilie and Thompson, 2006) or increasing its tempo (Dalla Bella et al., 2001) cause musical passages to sound happier. Additionally, listeners tend to choose higher pitch heights, faster tempi, and major modalities when crafting melodies intended to sound happy rather than sad (Quinto and Thompson, 2012). Although these studies provide useful insight into the effects of experimentally manipulating pitch and timing, they are not able to comment on musicians' artistic decisions. Consequently the following studies explore how composers naturally sculpt relationships between pitch, timing, and modality to convey emotional messages, offering new insight into the complex relationship between instrumental constraints, compositional structure, and musical communication.

## Instrumental constraints and emotional messages

Although cues such as pitch height and timing play a powerful role in conveying emotion across a range of music, they are not equally available on all instruments. For example, in a Saturday Night Live skit comedian/musician Steve Martin memorably poked fun at the banjo's inability to play sad music. His humorous struggle to pluck a convincing tune about “murder, and death, and grief, and sorrow” aptly illustrates the challenge of playing depressing music on a decidedly happy instrument (Martin, 1976). Entertainment aside, Martin's monolog raises a musically interesting and psychologically important question: are some instruments “emotionally typecast” and unable to break beyond a narrowly defined **emotional palate**?

KEY CONCEPT 2 **Emotional palate (for musical instruments).**

An instrument's acoustic constraints narrow the ranges of emotions that can be conveyed. For example, the challenges in producing low pitched, slow moving melodies on the xylophone and banjo renders them ill-suited for conveying sadness.

Passages on the banjo are generally high in pitch, and played on wire strings (Stephey and Moore, 2008) ringing for short durations—discouraging performers from playing slow melodies (hence the classic banjo “finger picking” sound). Although **acoustic constraints** on playing low and slow are not problematic for signaling happiness, they pose significant barriers to conveying sadness. As formally testing the limits of any single instrument is challenging, my colleagues and I explored the issue of instrumental constraints through a comparative analysis of repertoire for two acoustically similar instruments—the xylophone and marimba (Schutz et al., 2008). The degree to which they share key properties while differing in their ability to produce cues for sadness makes them well suited for exploring the effects of instrumental constraints on emotional communication.

KEY CONCEPT 3 **Acoustic constraints (on musical instruments).**

An instrument's design results in both constraints and affordances. The xylophone and banjo are restricted to relatively high pitches, as they cannot produce notes below the xylophone's lowest bar, or the banjo's lowest string. Similarly the tuning of intervals on fixed pitch instruments is inflexible—regardless of the notes' musical functions.

The marimba and xylophone are essentially “musical cousins.” Professional training for percussionists typically includes both instruments—reducing the chance that repertoire differences stem from performers' technical abilities. Crucially for our purposes, these instruments share many acoustic properties while differing in constraints on pitch and timing. Both are wooden bar percussion instruments struck with mallets, however the modern concert marimba spans a wide pitch range (typically 5-octaves), in contrast to the xylophone's narrower and higher pitch range (its lowest note is typically close to the marimba's mid-range). The marimba's capacity for sustain affords either slow, fluid passages or rapid melodies. In contrast, comparatively thick xylophone bars lack such resonance, encouraging the use of many notes per second to avoid sounding sparse. Consequently the marimba and xylophone differ sharply in their constraints with respect to pitch and timing, with the xylophone (similar to the banjo) restricted from playing low and slow—undermining its ability to convey sadness.

### Selection of repertoire

In order to select a repertoire of established literature we drew upon recital programs from a database maintained by the Percussive Arts Society, compiling a list of solos written for the marimba (36) and xylophone (13) by 31 different composers. Analyzing the beginning and ending of each movement (i.e., structurally distinct sections) separately, our data included 152 examples (32 xylophone, 120 marimba). Rather than a fully randomized sample, this approach prioritized the most frequently performed pieces, reflecting the input of two musical groups—composers and performers. The resulting list of pieces represented some of the most commonly performed solos, and two of the paper's co-authors (myself and Kristopher Keeton) who are professional percussionists/educators had personally performed or taught over 70% of this repertoire.

### Modality as a quantifier

Previous research has used modality as an *a priori* classifier (Huron, 2008; Turner and Huron, 2008; Ladinig and Huron, 2010) of emotion. **Modality** is a powerful cue for Western-acculturated listeners, with the major mode frequently regarded as “sounding happy” and the minor mode as “sounding sad.” Not only are major key melodies generally perceived as “more positive” by Western listeners (Gerardi and Gerken, 1995), minor key melodies tend to “sound happier” when altered to major (Gagnon and Peretz, 2003). Mode's importance has long been recognized (Hevner, 1935; Rigg, 1937), and often outweighs other cues such as pitch and timing (Eerola et al., 2013). This association is so pervasive that mode has even been used for psychological assessments of clinical deficits in music processing (Peretz et al., 1998), mood induction (Västfjäll, 2002; Houston and Haddock, 2007; Hunter et al., 2011), and assessing vision's influence on auditory judgments of interval affect (Thompson et al., 2008).

KEY CONCEPT 4 **Musical modality.**

The specific selection of pitches within a passage is governed by numerous factors, including musical mode. This refers to the “type of key” employed—with the most common being “major” and “minor.” Scales in minor keys have lowered third, sixth, and seventh scale degrees relative to major keys.

Admittedly, despite modality's power, its emotional control is not absolute. Much as master chefs mix ingredients to delight diners with desserts containing both salty and sweet components, composers can mix cues to provide nuance with respect to emotional messages (Hunter et al., 2008). Fast minor key themes such as the opening of Mozart's G minor Symphony (No. 40) do not strike most listeners as sad, as it moves quickly and uses relatively high pitches. However, these exceptions do not invalidate the broad recognition of modality's emotional power (Hevner, 1935; Rigg, 1937; Gerardi and Gerken, 1995; Gagnon and Peretz, 2003), reflecting the strong association between **modality and emotion**.

KEY CONCEPT 5 **Modality and emotion.**

Major key pieces tend to sound “happier” than minor for Western-acculturated listeners. Modality is one cue of many controlling emotion, and composers can balance other cues to avoid minor key pieces always sounding “sad” should they so desire. Nonetheless, modality is generally a powerful predictor of perceived affect.

Previous explorations of musical corpora found mode a useful *a priori* classifier. For example, Huron (2008) compared the average pitch height for major and minor key themes (i.e., well known monophonic melodies without harmonic accompaniment), finding the major to be 1.1 semi-tones higher. Such comparisons have also shown major key pieces exhibit louder dynamic markings than minor (Turner and Huron, 2008; Ladinig and Huron, 2010). These comparisons of monophonic melodies (absent accompanying voices) and isolated dynamic markings provide a useful parallel to observations that happy speech is higher in pitch and louder than sad speech (Scherer, 2003). Yet they do not address the question raised by Martin's “sad banjo” sketch—how instrumental constraints on the use of pitch and timing shape compositional decisions.

### Results of surveying repertoire for marimba and xylophone

**Figure 1:**
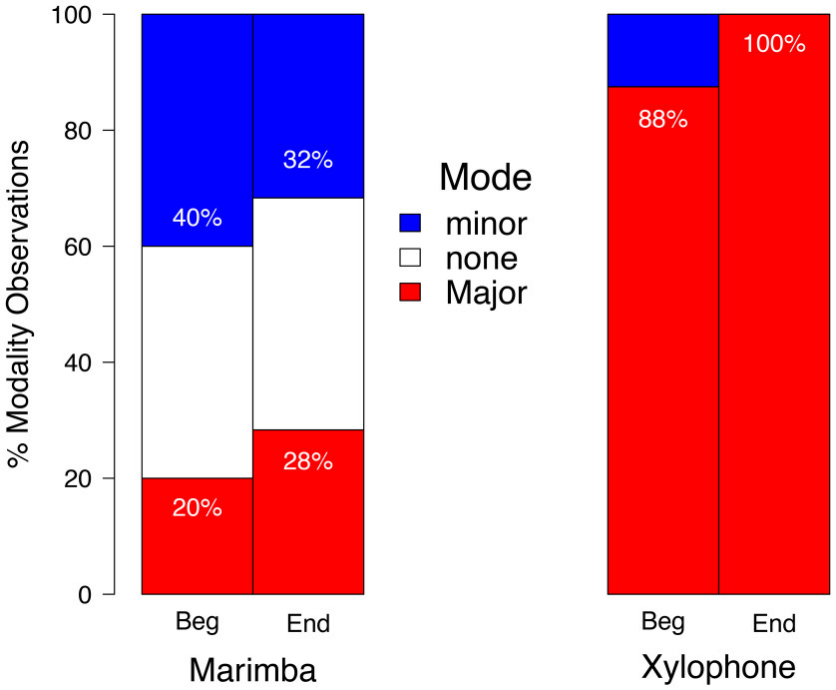
Literature frequently played on the xylophone rarely exhibits minor keys. This sample contains frequent use of the minor mode for marimba, with 60% of the tonal pieces exhibiting minor keys (ignoring marimba solos with no tonal center). In contrast it contains few instances of minor key xylophone pieces, which appear only twice within this survey.

Tabulating the relative proportion of modalities at the start and conclusion of our sample revealed that 60% of marimba pieces with clear tonal centers exhibited minor keys (Schutz et al., 2008). This provided a useful baseline for understanding composers' choices regarding modality when writing for a tuned, wood-bar percussion instrument offering a wide range of pitch heights and timings. In contrast, composers within this sample appear to avoid minor keys when writing for the xylophone, employing them in only 6% of the total analyzed repertoire (i.e., two compositions). See Figure 1 for a summary of these findings. As this sample included only pieces drawn from recital programs (rather than an exhaustive list), further research would help clarify questions about the repertoire as a whole. Nonetheless, this sample favored only those pieces favored by the performers themselves—reflecting the complex interaction between the choices of composers and performers in the evolution of an instrument's repertoire.

Both the percussionist-authors conducting that study immediately recognized that the only two examples of minor key xylophone pieces encountered shared something in common: they are rarely played on the xylophone. Subsequent inquiry with the percussionists listed in each surveyed program revealed that every performance had in fact occurred on the marimba. Performers offered straightforward explanations for their decisions such as “I personally don't like the sound of this piece on xylophone” (the piece in question is titled “Sonata for Xylophone”). When asked about “Fantasy on Japanese Woodprints”—a popular xylophone composition by Hovhaness (1965/1990) opening in minor—an accomplished percussionist replied “I've always thought this piece sounded better on marimba…in fact, I can't ever remember hearing a performance on the xylophone! I wish I had something more profound to tell you, but it's that simple” (Schutz et al., 2008). Personally, I found this unexpected outcome both intriguing and amusing. For despite many years of moving between the concert hall and research lab as a percussionist/researcher, I had never wondered *why* this xylophone repertoire seemed better suited for the marimba. This illustrates the symbiotic relationship between musical research and performance—performance related questions can lead to novel research discoveries, which can in turn inform both academic and artistic pursuits.

This survey of percussion repertoire is consistent with the idea that the xylophone's acoustic restrictions constrain its ability to convey sadness. Not only does this sample contain few instances of minor key pieces, performers demonstrate passive (albeit subliminal) resistance by regularly choosing to perform minor key xylophone solos on the marimba. The xylophone's pitch and timing constraints render it an inherently happy instrument. As illustrated by Steve Martin's performance and Willie Nelson's “You Just Can't Play a Sad Song on a Banjo” (Buskirk and Jackson, 1994), this challenge is not unique to the xylophone. The finding that composers shy away from using the minor modality when writing for instruments ill-suited to playing low and slow leads naturally to another question: Would composers also choose to write higher and faster passages when writing in major keys vs. minor keys?

## Modality constraints shape composers' use of pitch and timing

Building on this analysis of recital programs, I worked with undergraduate pianist Matthew Poon to explore whether composers choose different pitch and timing cues when writing in major vs. minor modes (Poon and Schutz, 2015). In other words, given that instrumental restrictions on pitch and timing shape composers' modality choices, do restrictions on modality in turn shape composers' crafting of musical passages? This reverses the question of how instrumental constraints on pitch and timing affect composers' selection of modality by analyzing how constraints on modality affect composers' selections of pitches and rhythms.

### Choosing an appropriate corpus

Our first challenge came in identifying repertoire with equal numbers of major and minor key pieces. We initially hoped to use sets of well-known themes, either from catalogs such as Barlow and Morgenstern (1983), symphonic themes from particular composer(s), or a fixed set of pieces (e.g., Haydn string quartets). However, balancing modality in those contexts quickly proved intractable, as Western music is overwhelmingly major. For example, only two of Mozart's 40+ symphonies are minor (Tan et al., 2010, p. 251), as are only 10 of Haydn's 100+ symphonies (Smith, 2002, p. 29). This same major-bias can be found in rock music (Temperley and de Clercq, 2013) as well as jazz^1^. Because modality plays a crucial role in emotional communication within music (Hevner, 1935; Gerardi and Gerken, 1995; Gagnon and Peretz, 2003; Eerola and Vuoskoski, 2013), this imbalance poses significant challenges to undertaking corpus analyses of **natural music** paralleling those of natural speech (Banse and Scherer, 1996; Sobin and Alpert, 1999). Fortunately, Johann Sebastian Bach (1685–1750) inadvertently provided a beautiful solution to this vexing problem—albeit in response to a different challenge.

KEY CONCEPT 6 **Natural music.**

Music by highly regarded composers, unaltered from the original pitches and rhythms. Its nuances pose certain challenges for scientific explorations, including correlations between pitches, rhythms, and modality. Nonetheless music that has stood the test of time holds strong ecological validity, and provides a useful parallel to “natural speech.”

Prior to Bach, Western instruments commonly used “just intonation”—a tuning system with roots extending back to ancient Greece (Wild and Schubert, 2008). Just intonation offers the most pleasing tuning of certain intervals; however, it is highly restrictive with respect to modulation (i.e., movement between keys). Although singers, violinists, and wind players can dynamically adjust tunings to alter problematic intervals, fixed pitch instruments such as the harpsichord and organ lack this capability. Just intonation limits fixed pitch instruments' ability to modulate, constraining composers' creative options. The “well tempered” approach gaining popularity during Bach's time loosened this constraint, affording new musical possibilities for fixed pitch instruments.

In order to take advantage of the compositional flexibility afforded by **well temperament**, Bach wrote 24 preludes and 24 fugues balanced in both **musical key and musical mode**^2^. Studied and performed widely by musicians ever since, this landmark collection of pieces inspired musical successors such as Frederic Chopin (1810–1849), whose *24 Preludes* use a similar organizational approach. We analyzed both sets to gain a broader and more generalized perspective, contributing to a growing body of research documenting systematic differences in low-level cues across musical eras and between geographic regions (Daniele and Patel, 2013, 2015).

KEY CONCEPT 7 **Well temperament.**

Removes constraints to modulation (i.e., changing keys) found in earlier tuning systems. This approach was especially welcome for fixed-pitch instruments such as the piano (and it's historical pre-cursors) as their tuning cannot be adjusted during the course of a performance.

KEY CONCEPT 8 **Musical key and musical mode.**

A composition's key type contains two components. The key “chroma” or name (e.g., “C”,“D”,“E”) and mode (major/minor). Pieces in C major vs. C minor will share a tonic (i.e., pitch home) of C, but exhibit different modalities. Conversely, pieces in D minor and B minor share a modality, but exhibit different tonic notes.

This corpus analysis of modality-balanced music widely recognized for its artistic and pedagogical value provides a useful counterpart to existing corpus analyses of natural speech. For although such analyses have proven insightful in linguistics (Banse and Scherer, 1996; Sobin and Alpert, 1999), efforts to conduct parallel musical corpus analyses have been complicated by two factors. First, although many adults are capable of generating sentences with different emotional connotations, few are capable of generating similarly effective musical passages. Second, those able to create effective musical compositions have historically chosen a disproportionate focus on major vs. minor keys. As modality plays a powerful role in conveying emotion, this imbalance across a range of musical styles presents significant challenges for identifying proper musical corpora (speech does not use modality, freeing linguists from this problematic challenge). Therefore, analysis of two 24 piece sets by renowned composers working in distinct musical eras offers a robust exploration of cues used for emotion in music, providing a useful musical counterpart to previous corpus analyses of speech.

### Exploring pitch and timing in keyboard music

Music for keyboard instruments^3^ such as the piano, harpsichord, or clavichord is ideally suited for our score-based corpus analysis. In contrast to the banjo and xylophone, the piano offers a wide range of pitches, giving composers numerous melodic and harmonic options—all of which can be precisely notated. Similarly, the instrument's range of note lengths affords fast-moving gestures as well as slow, evocative passages. Timing on such instruments is similarly amenable to precise quantification. In contrast to this wide range of pitch and timing possibilities, keyboard instruments are relatively constrained with respect to timbre variations for individual notes—at least relative to wind and string instruments. For example, Hugo Cole noted that “the timbre of a single oboe note may be varied in 98 ways by the use of different fingerings and methods of blowing” when discussing the challenges of notation (Cole, 1974, p. 128). These myriad timbral nuances are both difficult to discern from a notated musical score and variable between performers. As keyboard instruments produce sound from hammers striking (or plucking) strings whose movement trajectories are not readily altered, their timbre is less easily varied. Consequently keyboard literature is ideally suited for score-based corpus analyses as difficult-to-notate cues such as timbre are relatively constant, while composers have great latitude in selecting pitches and timings—cues that can be precisely notated.

Although quantifying pitch and timing in complex polyphonic music presents challenges, it offers four important benefits. First, musical scores clearly distinguish intention independent of production (which in speech might be influenced by other factors such as physiological constraints). Second, musical instruments' wide range of affordances provides a useful extension of speech utterances constrained by the vocal tract's physiology. Third, the notation of information from time periods prior to the advent of recording technology offers the possibility of exploring changes in cue use over centuries predating recorded sound. Finally, the structural approach of using all 24 keys within a single set of pieces offers a rare opportunity to explore the “top down” effects of modality. Bach and Chopin set out with the *a priori* intention to write a set of pieces balanced in modality—selecting pitch and timing cues fitting within each piece's pre-defined mode. Consequently, these sets offer a rare glimpse into how modality restrictions affect composers' selections of pitches and rhythms, complementing the first experiment examining how restrictions in pitches and rhythms shaped composers' selections of modality (Schutz et al., 2008). Basing this exploration on natural music means its outcomes will offer a useful counterpart to previous explorations of emotional cues in natural speech (Banse and Scherer, 1996; Sobin and Alpert, 1999).

### Cue extraction: operationalizing pitch height and attack rate

Our approach established the “center of gravity” of pitch height in each measure by weighting the pitch height of each note in accordance with its duration. Faithfully reflecting the nuances so important to these compositions led to challenges regarding treatment of grace notes, ornaments, double stemmed notes, and other such details common in music composed for artistic fulfillment, rather than scientific study. Although the proper way of treating such cases might garner some debate, they appeared in similar proportions amongst major and minor key pieces. Therefore, different decisions on their treatment did not affect the relative differences between major and minor pieces crucial to our analysis (further details can be found in Poon and Schutz, 2015).

We operationalized timing using attack rate—essentially the number of notes per second^4^—which offers several advantages over tempo. Primarily, it more closely parallels speech researchers' use of articulation rate, which is generally measured in the number of attacks^5^ per second (Johnstone and Scherer, 2000; Scherer, 2003). Occasionally called attack density (Pigeon and Verlinde, 2009; Mailman, 2012) or event density (Madison et al., 2011; Schaefer et al., 2011), this measure differentiates between passages with different rhythms played at identical tempi. For example, although the assessed segment of Bach's *F# minor Prelude* (No. 14) is marked at the same tempo of ♩ = 108 as the *Ab Major Prelude* (No. 17), its attack rate is considerably faster due to its denser rhythmic structure. However, despite a tempo marking of 108, its attack rate is considerably slower than Bach's *Bb Major Prelude* (No. 21), with a tempo of ♩ = 76.

We counted the number of note attacks per measure, treating simultaneous onsets such as chords as a single attack. We considered ornamentations to “decorate” individual notes, and did not alter the attack rate of measures with trills from the literal note durations specified in the score (although we counted grace notes marked explicitly). Using the tempo markings provided by Hans Bischoff Bach (1883), we then translated our tabulation of note-attacks-per-measure into note-attacks-per-second, allowing for differentiation between **attack rate and tempo**.

KEY CONCEPT 9 **Attack rate and tempo.**

Although correlated, tempo alone is not as sensitive to musical differences within or between compositions. Pieces with identical notated tempi have very different attack rates, and vice-versa. Attack rate is a closer parallel of the way timing is operationalized in speech. It is also more sensitive to measure-by-measure fluctuations in timing.

## Results and discussion

**Figure 2:**
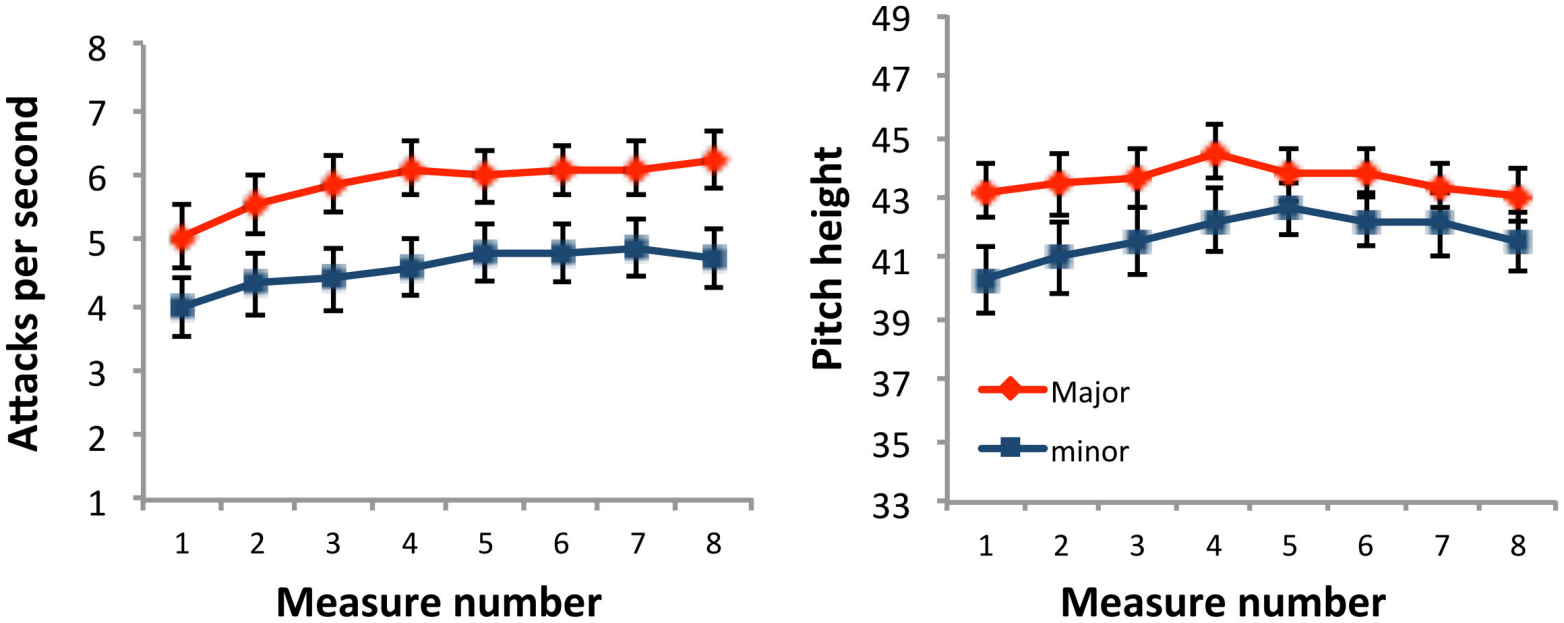
Major key pieces are higher and faster than minor key pieces. The 36 major key pieces exhibited faster rates of attack **(left)** and higher pitch heights **(right)** in major vs. minor keys. Data are displayed for the first eight measures of each piece. Error bars represent 1 standard error about the mean.

The most important outcome is finding that this corpus of unaltered music by renowned composers employs musical cues for emotion in a manner broadly consistent with those found in speech (Figure 2). On average, the 36 major key pieces are both significantly higher and faster. Specifically, major pieces are approximately two semi-tones higher in pitch (E4 vs. D4), and 29% faster in timing (5.85 vs. 4.56 attacks-per-second). Previous studies explored the effects of altering melodies to use higher pitches (Hevner, 1937; Ilie and Thompson, 2006) and/or faster timings (Dalla Bella et al., 2001). Here, we complement those by demonstrating that Bach and Chopin *chose to use* higher pitch heights and faster timings for major vs. minor pieces. Although our analyses consisted of only 72 pieces, they are balanced across numerous crucial parameters: modality, key chroma, and consistent within other important parameters such as composer and time period (i.e., all pieces came from single sets composed in a relatively short time span).

These results extend previous studies demonstrating differences between sets of major and minor key pieces, which often focus on either isolated musical properties such as individual dynamic markings (Turner and Huron, 2008; Ladinig and Huron, 2010), tempi (Post and Huron, 2009), or rhythmic variations of monophonic themes (Huron and Ollen, 2003; Patel and Daniele, 2003). Although studies of relationships between timing and pitch (Broze and Huron, 2013) and analysis of differences in pitch height between major and minor key themes (Huron, 2008) offer useful insight, they generally do not account for the rich musical material beyond monophonic melodies (i.e., harmony/accompaniment) found in most Western compositions. Therefore, to the best of our knowledge this study is the first analysis of both pitch and timing cues accounting for all voices including both harmonic and melodic content. Bach and Chopin wrote each set with a clear goal of using all 24 keys—creating a corpus balanced with respect to modality. Their selection of notes for major vs. minor pieces when writing for an instrument offering a wide range of options provides a useful opportunity to explore how low-level cues shared with speech (pitch, timing) are shaped by other music-specific decisions (i.e., modality).

**Figure 3:**
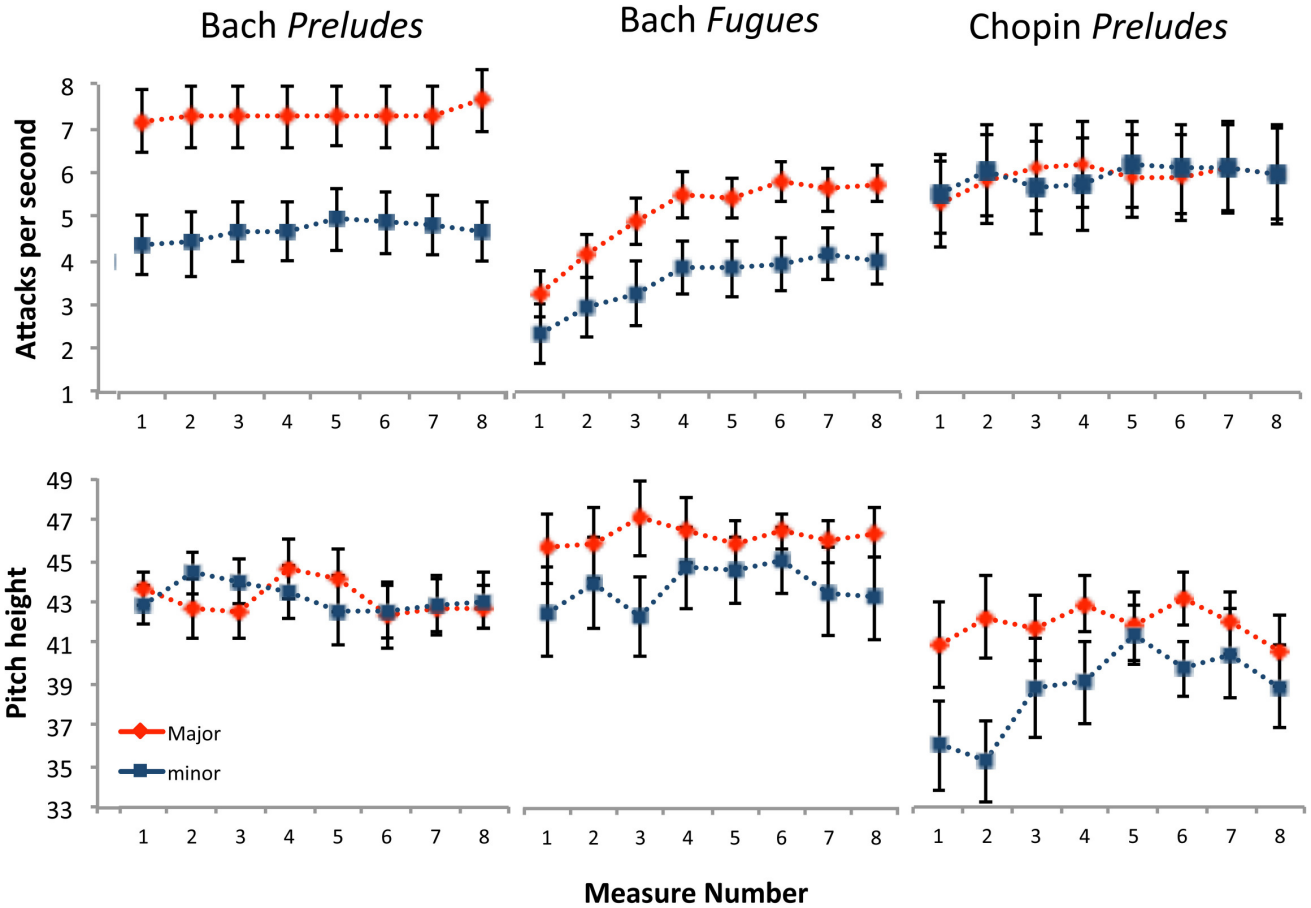
Trade-off of cues across the corpora. Bach's *Preludes* exhibited the largest timing difference between major and minor pieces, but the smallest pitch difference. Conversely, Chopin's *Preludes* exhibited the largest pitch difference but the smallest timing difference. Data are displayed for the first eight measures of each piece. Error bars represent 1 standard error about the mean.

Although the data are consistent with initial predictions that major key pieces would be higher and faster, further analyses of the individual corpora (Bach's *Preludes*, Bach's *Fugues*, and Chopin's *Preludes*) provide a more nuanced view raising intriguing questions. Bach's *Preludes* exhibit the largest difference in timing, yet the smallest difference in pitch height (Figure 3). Conversely, Chopin's *Preludes* exhibit the smallest difference in timing but the largest in pitch (Bach's *Fugues* shows moderate differences in both cues). Consequently these sets appear to “mix” cue differentiation—possibly in an attempt to add complexity and nuance to their compositions. Admittedly, the exploration is both *post-hoc* and limited in scope, and further research is needed to explore whether these are representative of larger trends. Nonetheless, they provide an intriguing opportunity to explore trade-offs in cue use within different musical eras, offering insight into historical changes. This is useful, given recent interest in how the nature of modality changed during the Romantic era, where minor keys took on a different “meaning.” During this period composers favored a style typically discussed as “passionate” and “emotional,” defying previous conventions by using major keys more frequently in tender/lyrical passages (Horn and Huron, 2015).

### Discerning the “proper” tempi in music by J.S. Bach

**Figure 4:**
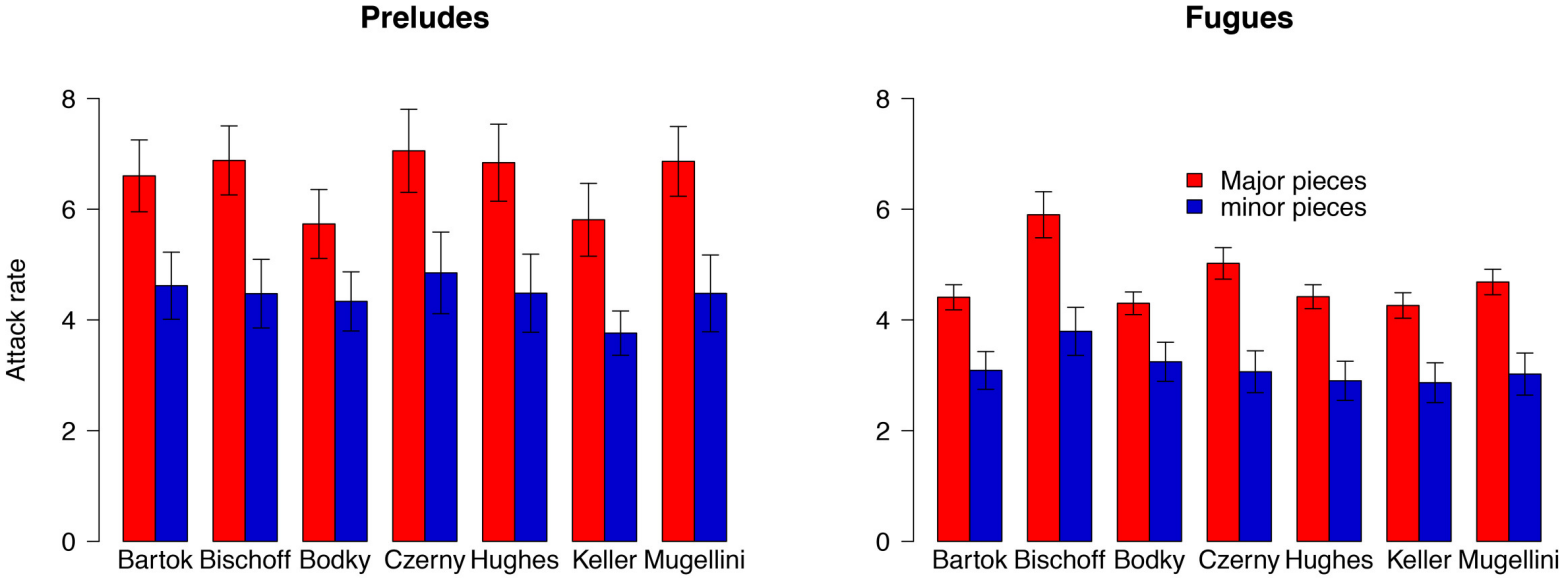
Major key pieces are faster than minor across all editorial tempi assessed. Differences in recommended tempi for individual pieces varies considerably for Bach's *Well Tempered Clavier*. Despite these differences, the difference in major vs. minor key attack rate is consistent amongst each of the 7 sets of editorial tempi outlined by Willard Palmer.

Bach did not give explicit tempi because the metronome did not achieve widespread use until after his death (Samama, 2016, p. 61). Therefore, our timing analysis used tempi suggestions provided by the respected editor and interpreter Hans Bischoff (Bach, 1883). As scholars often disagree about the correct tempi for these pieces, we assessed the stability of our findings using renowned musicologist Willard Palmer's (Bach, 2004) helpful table of seven different sets of tempi. Re-running our analysis, we found major pieces faster than minor *in each of these seven cases* (Figure 4). Therefore, we conclude that within this corpus Bach's major key pieces are faster than minor—regardless of which set of **editorial tempi** are consulted. As Palmer's data provide useful insight into an issue of broad relevance, we have created an interactive online tool for researchers, pianists, and music lovers to explore this landmark composition at www.maplelab.net/bachtempi.

KEY CONCEPT 10 **Editorial tempi.**

Widespread use of the metronome did not become common until after Bach's time. Consequently there is considerable debate over the “proper” tempi for many of his pieces, including those within the *Well Tempered Clavier*. As our findings held consistently across all seven editors and commentators, we conclude they generalize across different interpretations.

## General discussion

Music's powerful ability to convey and evoke emotion has long fascinated musicians (e.g., Bernstein, 1976), philosophers (e.g., Wittgenstein; Hagberg, 2008), and scientists (e.g., Darwin, 1872) alike. In fact, the birth of music cognition as a fully-fledged discipline traces in no small part to Leonard Meyer's seminal *Emotion and Meaning in Music* (Meyer, 1956). This interest has driven considerable research, with a survey noting 251 published studies of musical emotion over a 20 year period (Eerola and Vuoskoski, 2013). In order to avoid confounding variables, many studies of specific acoustic cues use stimuli either (a) composed wholly for scientific exploration (Thompson and Robitaille, 1992), (b) experimentally manipulated (Schellenberg et al., 2000; Ilie and Thompson, 2006), and/or (c) culturally unfamiliar single line melodies—or some combination of these techniques (Schellenberg et al., 2000). In contrast, the observational studies described here involving repertoire for the marimba and xylophone (Schutz et al., 2008) and the keyboard music of Bach and Chopin (Poon and Schutz, 2015) complement those approaches by focusing on acclaimed repertoire reflecting the decisions of highly trained musicians. In doing so this work strikes a balance between using ecologically meaningful material and systematically controlling multiple musical parameters—a challenge noted by Eerola and Vuoskoski (2013) after their important large-scale review of research on this topic.

These findings extend experimental work manipulating parameters such as modality, timing, and pitch height (Quinto and Thompson, 2012; Eerola et al., 2013), offering new musical perspective. Specifically, they document (1) composers avoid writing minor key works for instruments restricted from playing low pitches and slow rhythms^6^; and (2) when writing minor key pieces, Bach and Chopin selected lower pitches and slower rhythms than when writing major key pieces. This unorthodox approach of exploring these cues' natural use led to unanticipated findings that could spark fruitful future research projects, such as performer's resistance to composers' attempts to introduce minor key pieces to the xylophone repertoire, and the surprising consistency of major/minor timing differences in Bach despite considerable editorial disagreement regarding the correct tempi.

These results hold potentially useful insights pertinent to the emerging field of “critical organology,” which explores the relationship between instruments and their artistic roles/functions (Tresch and Dolan, 2013). Scholars in that discipline examine a variety of issues related to instruments and compositions, such as how the influence of string layout on a violin encourages certain types of melodies (De Souza, 2017). Findings that the acoustic structure of instruments shapes composers' choices of modality (Schutz et al., 2008), and that modality shapes composers' choices of pitches and rhythms (Poon and Schutz, 2015) complement existing approaches to critical organology by offering useful insight into the complex relationship between instrumental constraints, compositional choices, and the evolution of musical repertoire.

Finally, in addition to providing a novel corpus analysis of natural music complementing corpus analyses of natural speech, the approach of analyzing musical scores suggests possibilities for musical research beyond what is available to linguists—exploring the evolution of cues across centuries. As the widespread use of musical notation predates audio recording technology, score-based analyses of pitch and timing offer great potential for insights into changes in cue use over time periods that would be difficult for spoken utterances. This is also important for musical scholars, given increasing documentation of changes in low level patterns between musical eras (Daniele and Patel, 2013, 2015; Horn and Huron, 2015).

In conclusion, this *Focused Review* highlights connections between my team's original findings and other research disciplines. The studies described here provide a complementary perspective drawing upon the intuitions of highly trained musicians. Admittedly, exploring the natural use of cues and evolution of musical repertoire leads to numerous challenges, and this work raises as many questions as it has answered. However, my hope is that these results will suggest to others useful paths of future inquiry regarding music's profound ability to convey and evoke emotion—an issue of interest to anthropologists (Mithen, 2005), psychologists (Pinker, 2010), musicians (Juslin and Sloboda, 2010), and linguists (Fitch, 2010) alike.

## Author contributions

The author confirms being the sole contributor of this work and approved it for publication.

### Conflict of interest statement

The author declares that the research was conducted in the absence of any commercial or financial relationships that could be construed as a potential conflict of interest.

## Footnotes

^1^Shanahan, personal communication July 9, 2015. Based on post-publication insights to his corpus analysis of jazz harmony (Broze and Shanahan, 2013).

^2^He later wrote a second book containing an additional 48 Preludes and Fugues.

^3^Bach wrote the *Well Tempered Clavier* for a precursor to the modern piano. Although it is frequently played on piano, it can also be played on the harpsichord, organ, or various other instruments (including the marimba). For the sake of linguistic compactness I refer to “music for keyboard instruments,” differentiating it from music written for string or wind instruments.

^4^This method technically counted note attacks per second rather than individual notes (i.e., a chord with three simultaneous notes counted a single attack). For full details see Poon and Schutz (2015).

^5^Speech researchers often quantify this as “articulation rate”, however as articulation has a specific definition in the context of music we are intentionally using attack rate in this article to avoid confusion.

^6^The analysis of xylophone repertoire included only the most commonly performed pieces. It is possible that composers have written additional, lesser-performed minor key xylophone solos not captured by the survey.
